# Two-Stage Fungal Pre-Treatment for Improved Biogas Production from Sisal Leaf Decortication Residues

**DOI:** 10.3390/ijms10114805

**Published:** 2009-11-06

**Authors:** Mutemi Muthangya, Anthony Manoni Mshandete, Amelia Kajumulo Kivaisi

**Affiliations:** 1 Department of Biochemistry, School of Medicine, University of Nairobi P.O. Box 30197 Nairobi, Kenya; E-Mail: temi2m@yahoo.com (M.M.); 2 Department of Molecular Biology and Biotechnology, College of Natural and Applied Sciences, Uvumbuzi road, University of Dar es Salaam P.O. Box 35179, Dar es Salaam, Tanzania; E-Mail: akivaisi@amu.udsm.ac.tz (A.K.K.)

**Keywords:** two-stage pre-treatment, sisal leaf decortications residue, biological, fungi, lignocellulose, anaerobic digestion

## Abstract

Sisal leaf decortications residue (SLDR) is amongst the most abundant agro-industrial residues in Tanzania and is a good feedstock for biogas production. Pre-treatment of the residue prior to its anaerobic digestion (AD) was investigated using a two-stage pre-treatment approach with two fungal strains, CCHT-1 and *Trichoderma reesei* in succession in anaerobic batch bioreactors. AD of the pre-treated residue with CCTH-1 at 10% (wet weight inoculum/SLDR) inoculum concentration incubated for four days followed by incubation for eight days with 25% (wet weight inoculum/SLDR) of *T. reesei* gave a methane yield of 0.292 ± 0.04 m^3^ CH_4_/kg volatile solids (VS)_added_. On reversing the pre-treatment succession of the fungal inocula using the same parameters followed by AD, methane yield decreased by about 55%. Generally, an increment in the range of 30–101% in methane yield in comparison to the un-treated SLDR was obtained. The results confirmed the potential of CCHT-1 followed by *Trichoderma reesei* fungi pre-treatment prior to AD to achieve significant improvement in biogas production from SLDR.

## 1. Introduction

The sisal plant *Agave sisalana* Perrine, occupies the 6^th^ place among fibre plants, representing 2% of the world's production of plant fibres (plant fibres provide 65% of the world's fibres). However, sisal fibre production is a high waste industry currently using only about 2% of the plant as fibre and the rest being various residues, including sisal leaf decortications residues (SLDR), sisal short fibres and sisal stems. The traditional wet sisal leaf decortication process generates about 100 m^3^ and 25 tonnes of wastewater and solid residues, respectively, per tonne of sisal fibres produced. In Tanzania, it is projected that 100,000 tonnes of sisal fiber will be produced annually by the year 2010. This will result in generation of some 10 million m^3^ of sisal decortication wastewater and 2,500,000 tonnes of solid sisal decortication residues composed of about 2,000,000 tonnes of SLDR, the rest being short fibre residues. It has been previously estimated by Bradon [1] that the waste discharged during processing of 100 tons of sisal has a polluting character equivalent to sewage (PCES) of a population of between 22,000 and 33,000 people. With a projected production of 100,000 tons by 2010 it means a PCES of between 22,000,000 and 33,000,000 people. Although sisal waste is a menace to the environment, it also represents enormous underutilized bioresource for bioconversion into high value commodities such as animal feed, edible mushrooms, biofuels, biofertilizer etc. SLDR/sisal fibre residue can be efficiently exploited in a manner that is both profitable and sustainable under anaerobic digestion to produce biogas a renewable and environmental friendly form of energy [2,3]. Biogas can be used as vehicle fuel, for heating and for electricity production; hence the use of fossil fuel can be reduced enabling CO_2_-levels to be lowered in conformity with the Kyoto protocol [4]. To this effect the first sisal biogas plant in the world located at the Katani Ltd's estate at Hale, Tanga, for production of biogas, electricity and bio-fertilizer from SLDR at pilot scale was commissioned in the year 2007 in Tanzania. The sisal waste fed biogas plant is able to produce 300 kilowatts of electricity. The electricity is used mainly within the decortication plant and some of the excess can also be supplied to the domestic quarters within the estate. The excess biogas can also be distributed to surrounding communities to cover cooking and lighting requirements.

Anaerobic digestion occurs through the synergistic interaction of four different classes of microorganisms; hydrolytic, acidogenic, acidogenic and methanogenic bacteria in a multi-step process [5]. Hydrolysis has been found to be the rate-limiting step during AD of solid materials containing lignocellulose [6]. This limitation is mainly due to enzyme accessibility problems associated with the composition and structure of lignocellulosic substrates.

In cellulosic residues, the polysaccharides, cellulose and hemicellulose are intimately associated with lignin in the plant cell wall [7]. The lignin component acts as a physical barrier and must be removed to make the carbohydrates available for further transformation processes. Pre-treatment prior to AD has been proven to be one of simple and effective methods to improve biodegradability and biogas production from lignocellulosic materials [8] by promoting the hydrolysis step. Pre-treatment methods used include; physical/mechanical (*e.g.*, milling, grinding and irradiation), chemical (*e.g.*, alkali, dilute acid, oxidizing agents and organic solvents), physicochemical (*e.g.*, steam pre-treatment/autohydrolysis, hydrothermolysis and wet oxidation) and biological, or a combinations of these [2,9]. Biological pre-treatment methods have not been developed as extensively as physical-chemical methods for improving hydrolysis of lignocellulosic substrates. However, the former methods have the advantage that they are simple and do not require major capital investments [10]. In a recent investigation on the effect of pre-treatment of SLDR prior to AD using fungal strains; CCHT-1 and *T. reesei* separately, Muthangya *et al.* [11], obtained a 24–30% increment in methane yield compared to the untreated SLDR. Considering the biological activity nature of the strains used, further enhancement was anticipated if the two were combined. This study therefore determined the effect of pre-treating the SLDR prior to AD with the same fungal strains in succession on methane yield. Biogas manure (anaerobic digestate), has been reported [12] to be a suitable fertiliser for agricultural soil, since it contains organic matter and plant nutrients (N, P, K and Mg) which positively affect soil quality by improving the soil structure, increasing the water-holding capacity and stimulating microbial activity. The end result is not only an increase in soil quality, but also higher crop yields and better grain quality in comparison with unfertilised soil, or equivalent effects after application of artificial fertiliser. Anaerobic digestate obtained in this study was analysed for fertilizer qualities.

## 2. Experimental Section

### 2.1. Substrate and Anaerobic Digestion Inoculum

Sisal leaf decortication residues, a leafy biomass produced during sisal decortication were obtained from a sisal-processing factory at the Hale sisal estate, Tanga, Tanzania, while an active inoculum used in anaerobic digestion experiments was obtained from a 10 year old pilot batch manually stirred tank bioreactor, used for digesting SLDR at the same sisal estate. The inoculum was carried to the laboratory in twenty-five litre plastic containers with airtight lids. The compositions of the SLDR used in this study before and after pre-treatment are shown in Table 1. *Trichoderma reesei* QM-9414 spores in 20% glycerol were generously supplied by the Department of Biochemistry, Uppsala University (Sweden) while strain CCHT-1 was obtained from dumps of decomposing sisal decortication residues at Hale sisal estate, Tanga, Tanzania.

### 2.2. Fungal Inocula for Pre-treatment

A local white rot fungus and a standard fungal species *Trichoderma reesei* were used as inocula to treat the substrate prior to anaerobic digestion. Pure mycelium culture of CCHT-1 was established and maintained according to Dhouib *et al.* [13]. *Trichoderma reesei* QM-9414 spores were cultured on 5% potato dextrose medium (agar 15 g/L, dextrose 20 g/L, potato extract 4 g/L with a final pH of 5.6 ± 0.2 at 25 °C) for 7–10 days. Pure cultures of the two fungi were maintained on 2% malt extract agar slants at 4 °C and grown at 27 ± 1 °C on plates of the same medium for pre-treatment inoculum preparation. Microbial inoculum for the two species was prepared as described in the case of mushroom spawn production according to Stamets [14] using sterilized wheat grains and expanded on sisal fibre dusts (a waste from sisal fibres brushing) which was then used in pre-treatment of SLDR.

### 2.3. Bioreactors

Pre-treatment of SLDR with the two fungi was carried out by solid-state fermentation in bioreactors which consisted of rectangular plastic containers measuring 23 cm × 14 cm × 9 cm (length, width and height, respectively) [Cello® Domestoware (Mkate), Dar es Salaam, Tanzania]. A total of 136 aeration holes of 0.7 cm in diameter and 3 cm apart were made in all the sides. The anaerobic biodegradability of pre-treated SLDR and control (un-treated) were investigated in 0.5 L bioreactors consisting of wide mouth Erlenmeyer conical flasks as described by Mshandete *et al.* [15].

### 2.4. Pre-treatment of SLDR

This experiment was designed to investigate the effect of a two steps pre-treatment of SLDR using the two fungi in succession first with CCHT-1 followed by *T. reesei* and vice versa prior to AD. Different inoculum concentration and optima periods of four days and eight days with CCHT-1 and *T. reesei*, respectively, as previously determined [11] were applied. In the first step, five different inoculum concentrations of 5, 10, 25, 30, and 50% of CCHT-1 (inoculum/SLDR wet weight) were used to inoculate 450 g (wet weight) of fresh SDLR and incubated at ambient temperature of 28 ± 2 °C for 4 days. After the fourth day, the second step was done by inoculating the same SLDR with 25% *T. reesei* inoculum (inoculum/SLDR wet weight) and incubating for further eight days at ambient temperature of 28 ± 2 °C. The second experiment was done to investigate the effect of pre-treating the residue using the two fungi at the same inoculation rates of 5, 10, 25, 30, and 50% for *T. reesei* and incubated at ambient temperature of 28 ± 2 °C for eight days, after which the second step was done by inoculating the same SLDR with 10% of CCHT-1 inoculum (inoculum/SLDR wet weight. Analysis of the pre-treated SLDR was done and the pre-treated substrate loaded into the anaerobic batch bioreactors.

### 2.5. Anaerobic Digestion Experimental Set-Up

The anaerobic digestion experimental set-up for the pre-treated substrates consisted of 36 batch anaerobic bioreactors, which included 30 (experimental) bioreactors to digest the pre-treated SLDR. Two sets of control were employed, one set of triplicate bioreactors containing untreated SLDR was included to mimic the conventional method where no pre-treatment is done, and the second set of triplicate digester contained only the anaerobic inoculum. The biogas produced from the control was subtracted from that produced in the digesters containing the substrate (SLDR). The volume of anaerobic inoculum added to all digesters was kept constant at 200 mL (5.84 g VS). Each digester was fed with 5.84 g VS of the biologically pretreated substrate in the ratio of 1:1 ratio (substrate:inoculum). The bioreactors were kept at an ambient temperature of 28 ± 2 °C and shaken manually for one minute thrice daily to provide substrate mixing, the digesters were run for 42 days. Methane content was determined after every 48 hours prior to biogas volume measurement as described in the analytical section.

### 2.6. Analytical Methods

The composition of 5 mL samples of the biogas was estimated by the absorption of carbon dioxide and hydrogen sulphide in concentrated alkaline solution using serum bottles as described by Ergüder *et al.* [16] while the volume of biogas formed during the experiment was measured using a graduated 100 mL gas-tight plastic syringe with a sample lock according to Mshandete *et al.* [15] and the methane yield from the biologically pretreated SLDR was compared with untreated SLDR. The pH before and after anaerobic digestion of the biomass and effluents was determined using a pH 209 meter (Hanna instruments® USA). Total solids, volatile solids (TS, VS) and the ash content of the substrate and inoculum were determined by the oven-drying and ignition method, respectively according to standard methods [20]. Total carbon was estimated by the dry combustion method previously described by Allen (1989) [18]. Organic matter content of the SLDR was done by the dry combustion method previously described by Lyimo *et al.* [19]. The total fibres were determined by the permanganate method as Neutral detergent fibre (NDF) and Acid detergent fibre (ADF) according to the method of Goering and Van Soest [20]. Total nitrogen of SDLR before and after AD was determined by the Kjeldahl method. Analysis of ash mineral (K, Ca and Na) in anaerobic digested SLDR were analyzed using an atomic absorption spectrophotometer, while phosphorous was determined spectrophotometrically using the ascorbic acid method according to standard methods [17].

## 3. Results and Discussion

**Figure 1.**
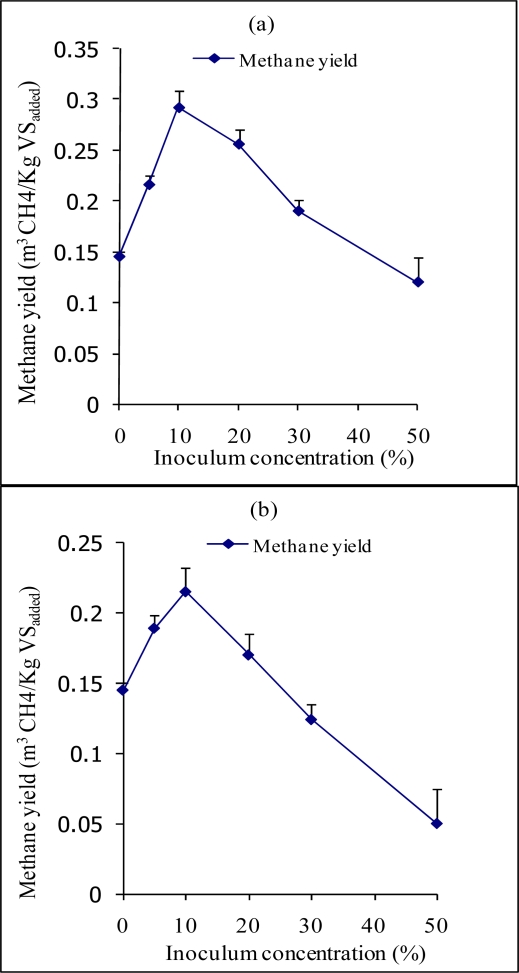
Methane yield of SLDR after two-steps pre-treatment with (a) 10% CCHT-1 inoculum followed by different *T. reesei* inocula concentrations (% wet weight of SLDR), (b) 25% *T. reesei* followed by different CCHT-1 inocula concentrations (% wet weight of SLDR).

Two steps biological pre-treatment of sisal SLDR prior to AD was investigated with two fungal strains in succession; CCHT-1 and *Trichoderma reesei* under solid state fermentation prior to AD, and the characteristics of the biofertiliser was determined. The untreated and biologically treated SLDR were anaerobically digested, and the daily biogas production for each run recorded. The methane yield, which was defined as the methane production per gram of VS added, was calculated and used to evaluate the effectiveness of biological pre-treatment. Optimum inoculum concentrations and incubation periods during pre-treatment were adapted to minimize cellulose degradation and make it more available for methane generation during AD of the pre-treated SLDR. The methane yield obtained after anaerobic digestion of the pre-treated SLDR ranged between 0.12 ± 0.03 and 0.292 ± 0.04 CH_4_ m^3^/Kg VS_added_. Pretreatment of SLDR with 10% of CCHT-1inoculum concentration for 4 days followed by different *T. reesei* inoculum concentrations for eight days (Figure 1a) enhanced the AD process with maximum methane yield of 0.292 ± 0.04 CH_4_ m^3^/Kg VS_added_ (101% methane yield increment compared to untreated SLDR). CCHT-1 grows naturally on sisal residues dumps; this observation possibly implies that, it is a good lignocellulosic degrader and hence was able to reduce the lignin and NDF contents by 16.5% and 22.5% (Table 1), respectively. Most probably, the removal of lignin reduced its sheathing of cellulose an observation in fungi pre-treatment of lignocellulosic materials reported by Hammel [21]. On further pre-treatment (second stage) of the same substrate with different *T. reesei* inoculum concentrations, which is a good producer of extracellular cellulolytic enzymes [22], prior to AD was possibly able to disrupt the crystalline structure of cellulose. The highest increase in cellulose content of about 21% was observed when 25% of *T. reesei* inoculum was used; this resulted in the highest in methane yield recorded. Further increase in *T. reesei* inoculum concentration resulted in decrease in cellulose content after pre-treatment as well in the methane yield during AD.

On the other hand, pretreatment of SLDR with 25% *T. reesei* inoculum concentration for eight days followed by different CCHT-1 inoculum concentrations for four days (Figure 1b) enhanced the AD process from 0.145 ± 0.01 CH_4_ m^3^/Kg VS_added_ in untreated SLDR to a maximum methane yield of 0.212 ± 0.02 m^3^/Kg VS_added_, corresponding to methane yield increment of 46%. Increase in the concentration of CCHT-1 beyond 10% during the pre-treatment process, resulted in decrease in cellulose content resulting in decreased methane yield. This is in agreement with the observation that, most fungi during bio-processing of plant material degrade lignin and cellulose simultaneously [23], thus the decrease in methane yield can be attributed to utilization of the carbon by the microorganisms during the pre-treatment, hence little substrate suitable for methane production was left [24]. This probably contributed to the lower methane yields observed in this study. A similar observation on the loss of methane yield with prolonged pre-treatment has been reported by Mshandete *et al.* [15]. Consequently, there is a an optimum incubation time after which the yield of digestible polysaccharides fails to increase further, or even declines, although lignin degradation continues achieving maximum digestibility.

During pre-treatment of SLDR, some of the large molecules were efficiently hydrolysed making them more easily available for AD an observation also reported by Mshandete *et al.* [15] working on sisal pulp pre-treated with activated sludge mixed culture under aerobic conditions. Pre-treatment of SLDR with CCHT-1 followed by *T. reesei* presents a viable process for generation of the carbohydrates in the biomass for biogas production in comparison to, pretreatment with *T. reesei* followed by CCHT-1 since higher methane yields were obtained in the former series.

Moreover, the final sludge is biologically stable and can serve as fertilizer or soil conditioner for agriculture. The biogas manure (biofertiliser) resulting from the digestate was obtained in 42 days, Mshandete *et al.* [3] reported 40 days of AD of aerobic pre-treated SLDR when stabilized organic rich manure a function of AD can be obtained, rich in the three major nutrients namely nitrogen, phosphorus and potassium. Analysis of the biofertiliser (Table 2) in comparison with the reported by Mshandete *et al.* [3] showed that the nutrients value was higher for two stage pre-treated substrate. The increase can be attributed to the pre-treatment using fungal inoculum which made much of the nutrients available in the sludge. The use of the biofertiliser leads to an increase in soil quality, higher crop yields and better grain quality in comparison with unfertilised soil, or equivalent effects after application of artificial fertilizer [25]. However despite the potential of the biofertiliser, it is subject to field trial and comparison to artificial fertilizers. However little information on biological pre-treatment of agro-residues is available for thorough comparison of the results obtained in this study.

## 4. Conclusions

The present study reports for the first time the two-stage fungi pre-treatment for enhanced biogas production from sisal leaf decortication residues. Studies on pre-treatment of lignocellulosic wastes are important in order to improve biogas production from the energy source rich cellulose and hemicellulose which are embedded in lignin. Thus, to metabolise the cellulose and hemicellulose contained in such materials lignin has to be, at least, partially degraded and the crystalline structure of cellulose disrupted. In the present study methane yield from SLDR in a two-stage pre-treatment approach using CCHT-1 followed by *T. reesei* was improved by 101% compared to the untreated control. The increase in methane yield was attributed to the improved biodegradability of SLDR after the pre-treatment process, which made more substrates available to be digested by anaerobic microorganisms. The results obtained in this work have clearly illustrated that there is a great potential of methane generation from enhanced anaerobic digestion of SLDR by biological pre-treatment using fungi. The findings from this study could be considered as a first step towards the development of strategies to stimulate hydrolysis further and ultimately increasing the methane yields from SLDR as the sole substrate. However, it remains to be tested in continuously stirred tank reactor before scaling up. Additionally, further research needs to be conducted to explore the mechanism of such improvement resulting from biological pre-treatment.

## Figures and Tables

**Figure 2.**
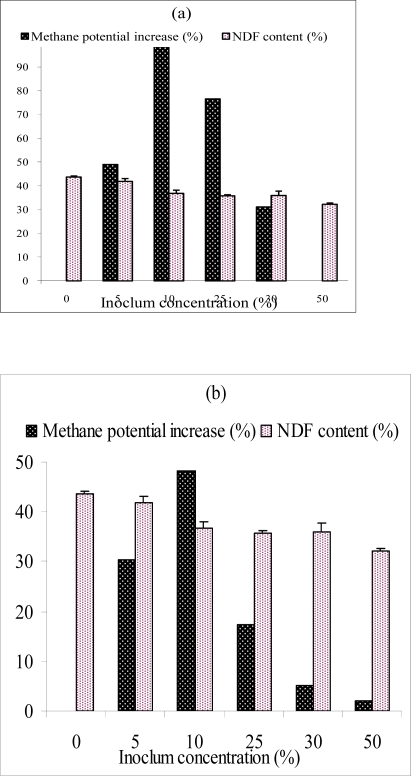
Methane yield and NDF content of SLDR after two-steps pre-treatment with (a) 10% CCHT-1 inoculum followed by different *T. reesei* inocula concentrations (% wet weight of SLDR), (b) 25% *T. reesei* followed by different CCHT-1 inocula concentrations (% wet weight of SLDR).

**Table 1.** Composition of SLDR before (untreated) and after two stage fungal pre-treatment (mean ± SD) n = 3.

**Determination**	**Untreated SLDR**	**Pre-treated (a)**	**Pre-treated (b)**
Total solids (TS)%	14.66 ± 0.14	11.19 ± 0.24	11.82 ± 1.19
Volatile solids (VS) (% of TS)	81.89 ± 2.67	82.21 ± 0.35	80.70 ± 2.01
Organic carbon^a^	48.19 ± 3.87	47.22 ± 3.93	48.82 ± 2.73
Neutral detergent fibres (NDF)^a^	44.5 ± 0.8	37.16 ± 1.65	36.00 ± 1.19
Acid detergent fibres (ADF)^a^	41.0 ± 0.7	29.2 ± 1.27	29.2 ± 1.27
Lignin^a^	7.2. ± 1.6	6.03 ± 0.42	6.73 ± 0.98
Cellulose^a^	64.1 ± 2.1	77.6 ± 3.6	66.5 ± 4.11
Hemicellulose^a^	3.5 ± 0.3	7.96 ± 0.31	4.96 ± 0.31

a ^%^of dry weight, Pretreated (a) CCHT-1 followed by *T. reesei* (b) *T. reesei* followed by CCHT-1.

**Table 2.** Comparison of biogas manure after AD of untreated SLDR and that pre-treated with 10% of CCHT-1 followed by 25% *T. reesei* inoculum, with that reported by Mshandete *et al.* [22]. (^a%^ of dry weight).

**Parameter**	**Untreated (mg/g)**	**Pre-treated (mg/g)**	**Mshandete *et al.* [3]**
Phosphorous	0.114	0.187	0.012
Sodium	15.7	19.5	1.44
Potassium	16.9	21.1	3.78
Total nitrogen^a^	1.46	1.54	0.01
Calcium	32.1	23.9	0.59
